# Cavitating Lesions around the Cochlea Can Affect Audiometric Threshold and Clinical Practice

**DOI:** 10.3390/audiolres13050072

**Published:** 2023-10-20

**Authors:** Giulia Zambonini, Sara Ghiselli, Giuseppe Di Trapani, Daria Salsi, Domenico Cuda

**Affiliations:** ENT Department, “Guglielmo da Saliceto” Hospital, 29121 Piacenza, Italy; g.zambonini@ausl.pc.it (G.Z.); g.ditrapani@ausl.pc.it (G.D.T.); d.salsi@ausl.pc.it (D.S.); d.cuda@ausl.pc.it (D.C.)

**Keywords:** third-window effect, otosclerosis, osteogenesis imperfecta, cavitating otosclerosis, cavitating osteogenesis imperfecta, pseudo-CHL, internal auditory canal diverticulum, “double ring” sign, cochlear implant

## Abstract

There are several pathologies that can change the anatomy of the otic capsule and that can distort the bone density of the bony structures of the inner ear, but otosclerosis is one of the most frequent. Similar behavior has been shown in patients affected by osteogenesis imperfecta (OI), a genetic disorder due to a mutation in the genes coding for type I (pro) collagen. In particular, we note that otosclerosis and OI can lead to bone resorption creating pericochlear cavitations in contact with the internal auditory canal (IAC). In this regard, we have collected five cases presenting this characteristic; their audiological data and clinical history were analyzed. This feature can be defined as a potential cause of a third-window effect, because it causes an energy loss during the transmission of sound waves from the oval window (OW) away from the basilar membrane.

## 1. Introduction

It is well known that mixed (MHL) and conductive hearing loss (CHL) can be associated not only with middle ear pathologies, but also with inner ear abnormalities that constitute the “pseudo-CHL”. This type of hearing loss (HL) is attributable to Third Window Syndrome. Different pathologies have been included in this syndrome being characterized by a common structural finding, that is an otic capsule defect that creates a “third window.” Consequently, this defect involves a similar spectrum of symptoms, signs (like dizziness and/or nystagmus) on physical examination and audiological diagnostic findings [1].

The most studied causes of Third Window Syndrome are the Minor Syndrome or canal dehiscence and enlarged vestibular or cochlear aqueduct. Less information is available about “pseudo-CHL”, which is related to bony changes in the otic capsule. 

Several disorders can damage the bony integrity of the inner ear, but the most common is probably otosclerosis. This pathology mainly affects the oval window (OW) in the early stages resulting in fixation of the stapedial footplate (fenestral otosclerosis), but may progress to involve the otic capsule (retrofenestral otosclerosis). The audiological finding is usually CHL, which may progress to MHL or sensorineural hearing loss (SNHL) [2].

Another condition causing similar symptoms is osteogenesis imperfecta (OI). OI is an inherited disorder of connective tissue metabolism that leads to bone fragility. OI is a heterogeneous disorder primarily caused by mutations in the genes involved in the production of type 1 collagen. 

Different types of OI classifications are used, but the most common types are based on clinical and radiological characteristics (Sillence classification), metabolic pathway and gene involvement [3].

The Sillence classification included only patients with defects in the primary structure of collagen and divides the disorder in 4 typologies: OI type I—autosomal dominant (AD) with blue sclerae, OI type II—perinatal lethal form with bone anomalies, OI type III—progressively deforming and OI type IV—AD with normal sclerae. Subsequently OI type V (with calcification in the intraosseous membranes) was introduced. Genetic classification is based on gene mutations, and, currently, XXII OI types are described [3]. In most cases, OI is correlated with a heterozygous mutation in the COL1A1 or COL1A2 genes. Both genes encode for type I (pro) collagen, a protein expressed in the extracellular matrix of bone, tendons, vessels, skin, sclerae, and blood [4]. OI patients have an increased susceptibility to fractures; other features include reduced body height, blue sclerae, poorly formed dentin, Marfan-like features, and early HL [5]. A large proportion of patients develop CHL between the second and fourth decades of life, which is associated with bony changes in the OW and results in stapedial fixation [6].

Bone damage caused by otosclerosis has been studied in the literature, and different patterns of bone demineralization have been described. The Authors’ intention is to focus on the pericochlear involvement and the extension to the anterior wall of the internal auditory canal (IAC), forming a cavity. This specific form of advanced otosclerosis has been defined as “cavitating otosclerosis” [7]. In these cases, the destruction caused by otosclerosis results in the formation of permeable luminal cavities containing fluid (cerebrospinal fluid, CSF). Several authors have associated this finding with a third-window effect, due to energy loss during the transmission of sound waves from the OW away from the basilar membrane [7,8].

This cavity, if particularly dilated, may communicate with the cochlear lumen, creating a direct connection between the CSF and the endolymph. In these cases, the HL appears as a “pseudo-CHL” for the third-window effect. This aspect is an important piece of information in the management of patients with pericochlear cavities in otosclerosis or in other diseases with bone damage of the otic capsule.

The pericochlear cavity has never been described in OI, but the presence of the third-window effect in OI cannot be excluded because of the similarity of the radiologic morphology as the presence of cavities and bone connections. 

In this study, we want to describe patients with pericochlear cavity to analyze the audiological characteristics and typology, the evolution of HL, and the treatments carried out to improve HL. It has been reported in the literature that when the bony damage is located in the anterior part of the labyrinth, patients generally develop a CHL/MHL without vestibular symptoms [9]. For these reasons, our hypothesis is that these patients may develop a pseudo-CHL that may also be related to a third-window effect, a defect in the bony structure of the otic capsule that locally reduces the hydrodynamic resistance of the perilymphatic space. 

## 2. Materials and Methods

A single-center retrospective analysis was conducted at the ENT department of “Guglielmo da Saliceto” hospital in Piacenza, Italy. Data from patients with HL who underwent CT (computed tomography) and MR (magnetic resonance imaging) of the temporal bone were retrospectively analysed. All patients were followed-up at the ENT Department of the Guglielmo da Saliceto Hospital in Piacenza from 1990 to the present day.

This research was registered with the Ethics Committee of the ‘Area Vasta’ Emilia-Nord under number 969/2022/OSS*/AUSLPC (date of approval 7 February 2023).

Adult patients (aged > 18 years old) affected by hearing loss and with radiological exams (CT and MR) who are referred to our department consecutively were included in this study.

Only subjects with the following radiological changes were included:

1. CT: osteorarefaction of the pericochlear area forming an ipodense cavity that communicates with the anterior wall of the internal auditory canal (IAC). It appears to be a large IAC diverticulum.
2. MR: a cavity with hyperintense signal, surrounding the cochlea and the IAC in a heavily T2-weighted sequence (MR cisternography). The cavity shows the same signal on the MR cisternography, it contains fluid with the same signal characteristics as the CSF.

We distinguished subjects with and without communication between the cavity and the bony labyrinth of the cochlea.

In these subjects, we evaluated some aspects of their medical history to understand the implications of these radiological findings:

- Etiology, the disease causing the bone remodelling;
- Age at diagnosis of HL;
- Type of HL at onset;
- Progression of HL;
- Stapes surgery, surgery performed/not performed and audiological outcome of the surgery.

Subjects under the age of 18, without radiological findings, hearing loss or who have not signed informed consent form were excluded from the study.

## 3. Results

We found five patients with pericochlear cavity (three females and two males). Overall, three patients were affected by otosclerosis and two by OI. One patient with otosclerosis had this abnormality in only one ear. The mean age at diagnosis of HL was 28 years (median 29; range 20–40 years). A progressive course of HL was observed in all cases (Table 1). The initial presentation of HL was different in our cases: five ears started with MHL, two with CHL and two with SNHL.

In the following paragraph, each clinical case is presented individually.

Patient #1, affected by OI. The patient reported that she had a fracture in her left arm as a child. Due to this incident and the presence of blue sclerae, she performed a genetic investigation with the finding of the COL1A1 gene mutation and diagnosis of OI type1.

She noted bilateral HL since the age of 20 with progression of MHL until stapes surgery was performed bilaterally. The right ear showed a good improvement in pure tone average (PTA) with complete closure of the air-bone gap (ABG). There were no surgical complications on this side. In the left ear, however, there was intraoperative bleeding during drilling of the ossified stapedial footplate, which necessitated reoperation for persistent HL. Resulting in a second revision surgery. Postoperative audiometric testing showed partial closure of the gap (ABG 20 dB).

**Figure 1:**
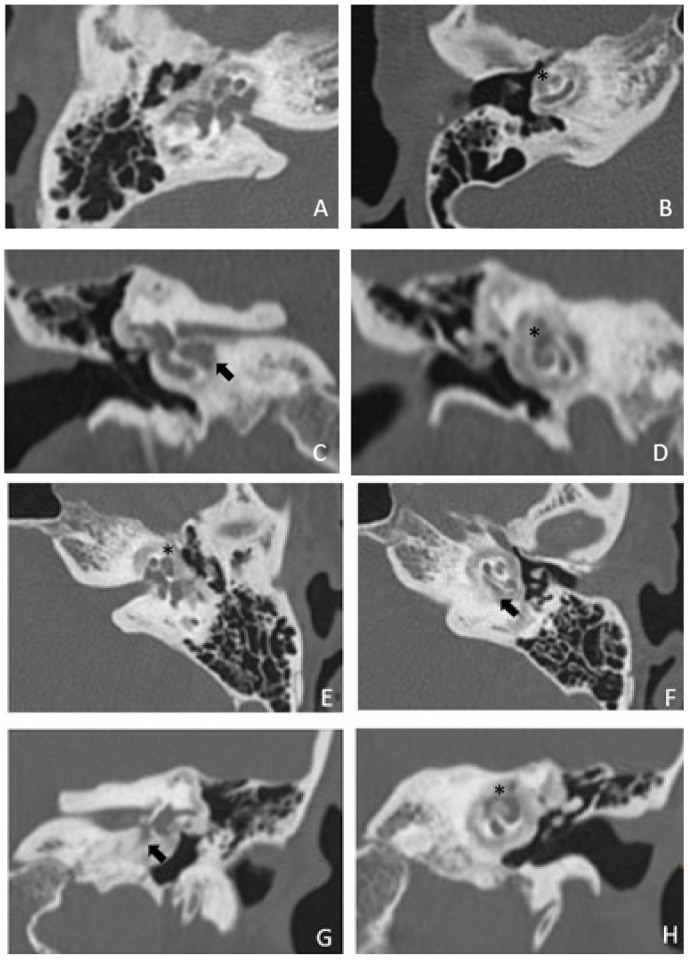
CT images of patient #1 with osteogenesis Imperfecta. (**A**,**B**), right side in axial plane. (**C**,**D**), right side in coronal plane. (**E**,**F**), left side in axial plane. (**G**,**H**), left side in coronal plane. A pericochlear cavity is visible in all figures; arrows indicate its origin from the IAC. Asterisks indicate extension of osteorarefation into the cochlea.

**Figure 2:**
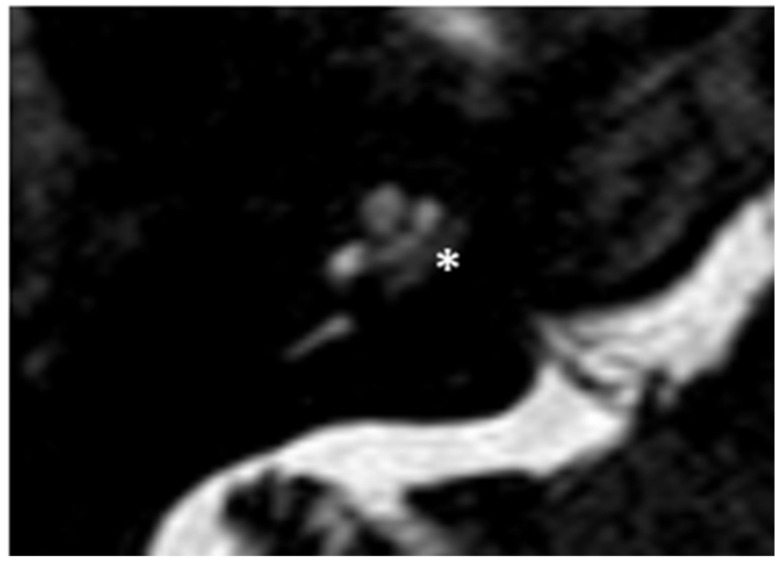
MR image of patient #1 with osteogenesis Imperfecta. MR cisternography showing the fluid-signal in the newly formed cavity (white asterisk).

Within a few years, both ears showed a worsening of bone threshold to SNHL bilaterally. In 2017 (27 years since diagnosis) she developed profound SNHL with left-sided anacusis and underwent cochlear implantation in the left ear. CT and MR images showed a large cavity around the basal turn of the cochlea and massive osteorarefaction around the cochlea (‘double ring’ sign). The cavity appeared to contain cerebrospinal fluid but did not communicate with the cochlear lumen (Figure 1 and Figure 2).

**Figure 3:**
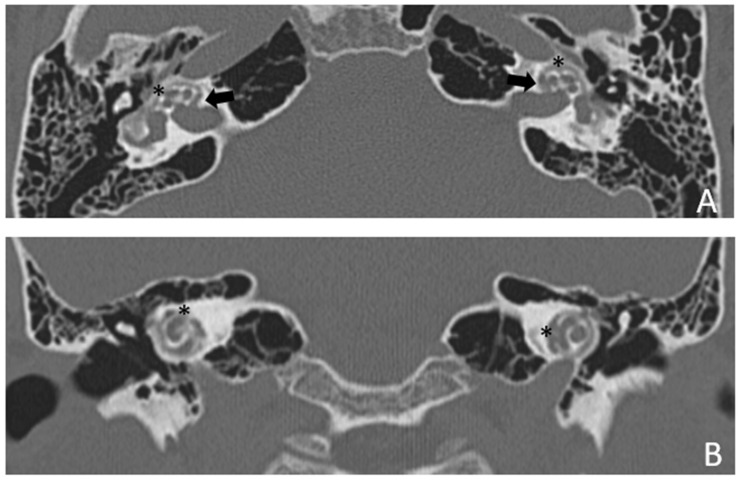
CT images of patient #2 with osteogenesis imperfecta. (**A**), axial plane; (**B**), coronal plane. A large pericochlear cavity is visible; arrows indicate its origin from the IAC, asterisks indicate the communication of the cavities with the cochlea.

**Figure 4:**
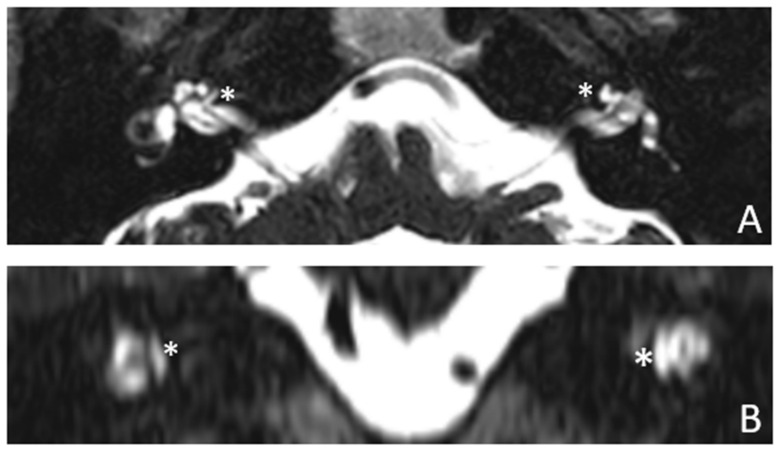
MR images of patient #2 with osteogenesis imperfecta. MR cisternography: (**A**), axial plane; (**B**), coronal plane. Note the fluid-signal in the newly formed cavities (white asterisk).

Patient #2, affected by OI-type I (COL1A1 mutation). MHL since diagnosis. Bone threshold was similar in both ears, but PTA was worse in the right ear with a larger ABG. HL progressed, but the difference between the two sides was maintained. The patient was fitted with conventional hearing aids bilaterally. Stapes surgery was not performed because of the radiological aspect (Figure 3 and Figure 4): a large cavity is present bilaterally and diffused communication between the cavity and the cochlea is evident.

**Figure 5:**
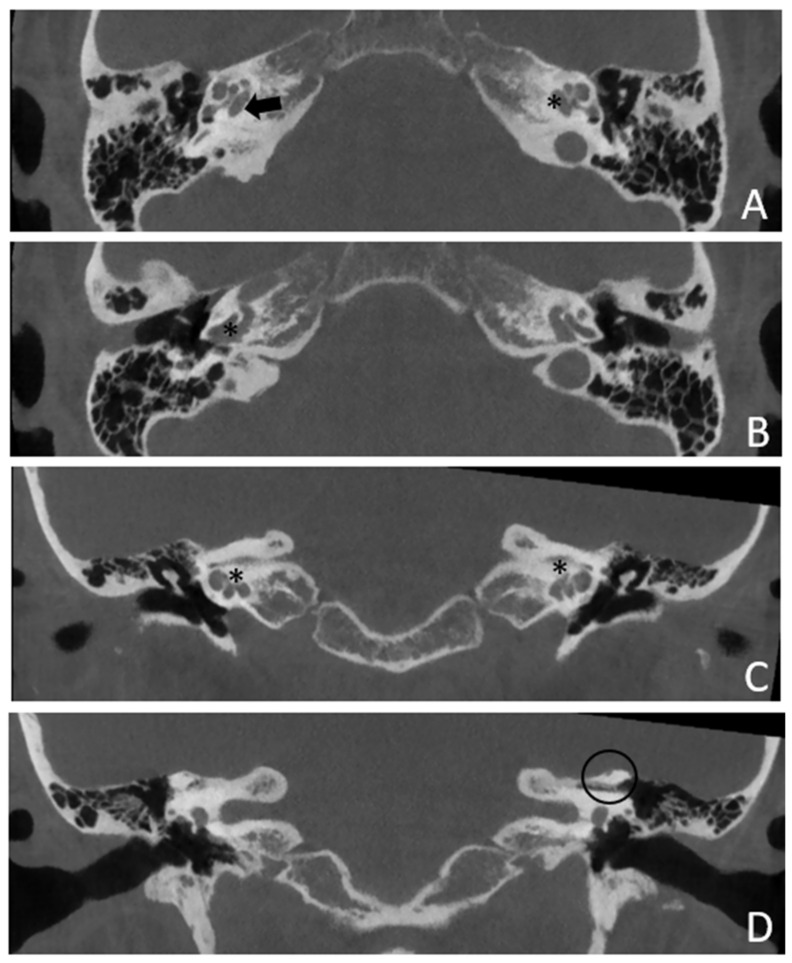
CT images of patient #3 with bilateral otosclerosis and SSCD of the left ear. (**A**,**B**), axial plane; (**C**,**D**), coronal plane. A cavitating osteorarefation around the cochlea is clearly visible. The communication of the cavity with the cochlea is indicated by asterisks. The arrow indicates the origin of the cavity from the IAC. The circle indicates the dehiscence of the SSC on the left side.

**Figure 6:**
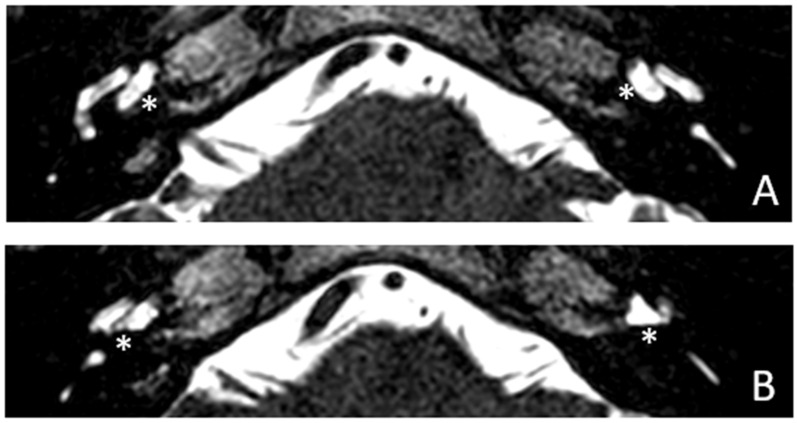
MR images of patient #3 with otosclerosis and SSC dehiscence on the left side. MR cisternography: (**A**,**B**), axial plane. Note the fluid-signal in the newly formed cavity (white asterisk).

Patient #3, affected by otosclerosis: HL started as mild CHL, but she had a progression with a worsening of the bone threshold, probably due to cochlear evolution of the disorder. Even in this case, there was no indication for stapes surgery because of the communication between CSF and perilymph through the cavities. The images also show a superior semicircular canal deiscence (SSCD)on the left. CT and MR images show the bilateral cavities (Figure 5 and Figure 6).

Patient #4, affected by otosclerosis: He developed mild SNHL from the age of 30 and progressed within twelve years to total deafness.

**Figure 7:**
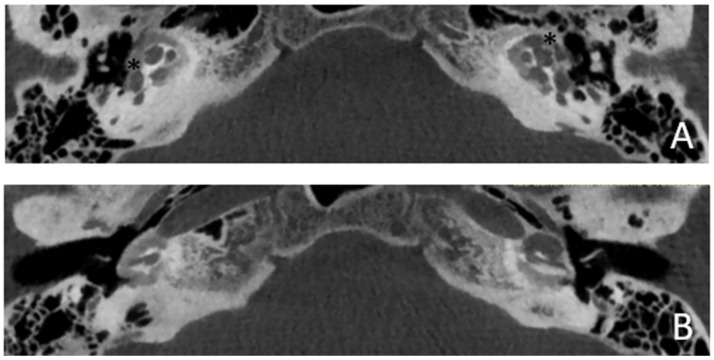
CT images of patient #4 with otosclerosis. (**A**,**B**), axial plane. The cavity around the cochlea is clearly visible as well as the communication of the cavitating osteorarefation with the cochlea, marked with asterisks.

**Figure 8:**
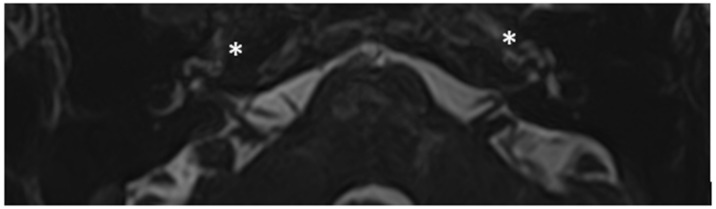
MR images of patient #4 with otosclerosis. MR cisternography, axial plane. Note the fluid signal in the newly formed cavity (white asterisks).

The patient underwent right cochlear implantation with excellent results. CT and MR images show a large empty space communicating with the cochlea bilaterally (Figure 7 and Figure 8).

**Figure 9:**
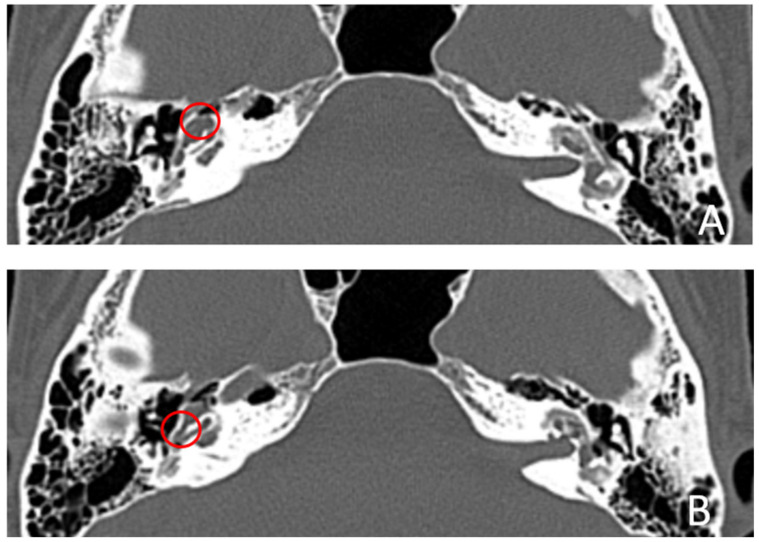
CT images of patient #5 with otosclerosis. (**A**,**B**), axial plane. The cavity around the cochlea is only visible on the right side (the circles indicate the contact of the osteorarefation with the cochlea), instead there is only an otosclerotic process without cavity on the left side.

**Figure 10:**
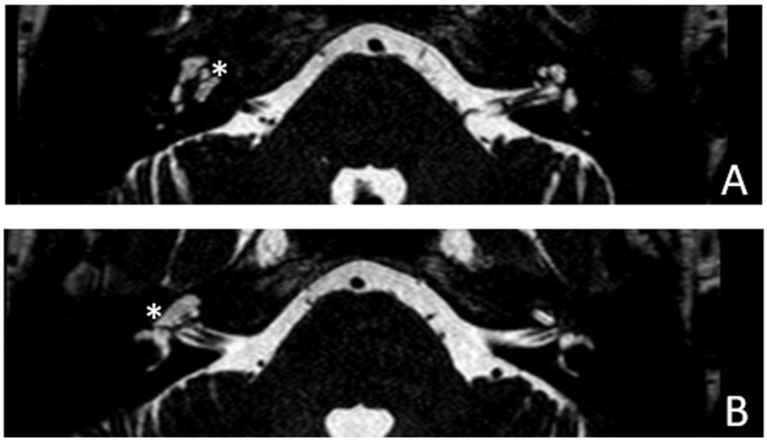
MR images of patient #5 with otosclerosis. MR cisternography. (**A**,**B**), axial plane. Note the presence of fluid signal in the newly formed cavity on the right side only (white asterisks).

Patient #5, affected by otosclerosis: She developed mild CHL on the right ear at the age of 29 and then mild CHL on the left side at the age of 36. On the right she progressed to moderate MHL, but on the left she progressed to severe SNHL. On CT and MR images (Figure 9 and Figure 10), the otosclerotic lesions are well visible bilaterally, but only the right ear was included in the study because a cavity around the cochlea, communicating with the perilymph, was present only on the right side. The patient received a left cochlear implant 10 years after diagnosis.

## 4. Discussion

This study was performed to test the hypothesis that patients with pericochlear cavities may develop pseudo-CHL possibly related to a third window syndrome.

In this study, we analyzed audiological characteristics of five patients affected by pericochlear cavity at the CT images in otoscleroris (in three cases) or OI (two patients) disease. We found a presence of MHL, CHL or SNHL at the time of the diagnosis with a progression of the degree of the HL over time. 

We suppose that these hearing disorders are related to the presence of pericochlear cavitary lesions. 

In fact, the literature agrees that not only middle ear disorders, but also pathologies related to bony changes in the otic capsule can be associated with CHL. These include otosclerosis and OI.

The HL due to otosclerosis is initially, usually associated with stiffness of the stapedial footplate (CHL), but it can progress to MHL and SNHL as the disorder spreads to the otic capsule. However, the mechanism of HL in OI is not clear and is likely to be multifactorial. OI may produce CHL due to a fixed and/or thick stape footplate and an accumulation of ossicular microfractures of unclear origin for the increased porosity and brittleness of the OI bone. SNHL in OI is associated with hyalinization and atrophy of the vascular striae, hair cell atrophy, and microfractures of the otic capsule [10]. Typically, HL is conductive in younger patients and progresses to sensorineural in older individuals. MHL often begins with fenestral damage causing CHL and progresses to retrofenestral elements causing SNHL [6,11,12].

In our sample, ABG is present at first diagnosis in four out of five patients; only one had SNHL from the beginning. Most likely, in this patient (patient #4) the otosclerosis started as a retrofenestral form and cochlear impairment was present since the beginning. The other patients had a standard course of pathology, accumulating cochlear damage over time, as evidenced by the CT and MRI images.

Certainly, both otosclerosis and OI can evolve and can cause retrofenestral areas of bone degeneration. The deformity may be very diffuse and may cause significant changes in the anatomy of the otic capsule. In general, the damage on CT of OI is earlier and more severe, bilateral, and symmetrical. Although the features of these two diseases are similar, histologically they differ in the bone damage: otosclerosis is limited to the endochondral layer of the otic capsule, instead OI involves the endosteum, the endochondral layer, and the periosteum [13,14].

A CT scan of the temporal bone can be used to assess bone degeneration. When the process develops around the cochlea in patients with otosclerosis, the so-called “double ring” sign is radiologically visible. This sign can also be seen in OI and is common (33.3%) [15,16]. Instead, the formation of an empty cavity around the cochlea that communicates with the IAC is defined as “cavitating otosclerosis” [7,17,18,19], but this peculiarity can also be reported in patients with OI. The cavity is formed by the union of the rarefaction around the cochlea and a diverticulum of the IAC. In these cases, MR is used to confirm the presence of CSF in the cavity. Consequently, in patients affected by otosclerosis or OI, CHL may develop not only because of the stiffness of the stape, but also by an alteration of cochlear fluid mechanics due to this newly formed cavity.

In addition, it is interesting to note that the coexistence of the IAC cavity and another type of third-window effect does not seem to add up in terms of the audiological features. In fact, in our patient #3, we found a SSCD in the left ear in addition to the otosclerotic cavity, but the ABG was only 10 dB on that side and progressed to 15 dB after 18 years (Table 1) whereas in the contralateral ear it was slightly higher both at first detection and later (15 and 25 dB). 

For better understand the nature of the hearing loss the acoustic reflex test can be carried out. In general, the acoustic reflex in CHL is an effective test to differentiate third-window disorders from middle ear diseases [20]. However, in the case of pericochlear cavitation, the stapedial stiffness and third window are likely to coexist and therefore the acoustic reflex is likely to be absent, as in the cases reported here. 

For these reasons, only radiological images can help identify these pathologies. It is important to note that when the aforementioned bony alterations are found on CT scan, it is necessary to investigate in detail in medical history concerning onset of hearing problem and typology of hearing loss. In particular, a differential diagnosis should be made with the perilymphatic fistula, an abnormal communication between the inner ear at the fissula ante fenestrum and surrounding structures. Cholesteatomas, head injuries and pathologies that lead to increased intracranial pressure may cause labyrinthine fistulas. The common symptomatology includes tinnitus, hearing impairment and vestibular symptoms [21].

To confirm the presence of a double etiology of CHL, some authors have studied patients with otosclerosis and concomitant third window after stapes surgery. In our sample, only one patient (patient #1) with OI underwent bilateral stapes surgery. She had different results on both sides despite similar radiological findings: she had an improvement on the right side, while an ABG remained on the left side. Both ears then deteriorated over time to SNHL.

In agreement with us, Ye Ji Shim et al., found that cochlear otosclerosis with cavitating lesions involving the IAC had significantly worse postoperative audiological outcomes than those without cavitating lesions [18]. They explain that the improvement in hearing is related to the resolution of the ossicular chain fixation, but the residual ABG is related to the third-window effect associated with the cavitating IAC diverticulum.

Stapes surgery shows the same audiological results in patients with otosclerosis and concomitant other types of third-window effect, such as superior semicircular canal dehiscence (SSCD) [22,23,24,25], enlarged vestibular aqueduct [23], and communication of the cochlea with the carotid canal [26]. Similar studies have not been performed for OI with third-window radiological features, but in general it can be said that stapes surgery has a low success rate (59.1%) in the OI population [10]. 

Based on the above literature, it can be hypothesized that the third-window effect, in our patient #1, was already present in the left ear at the time of stapes surgery and that the left CHL was at least in part a pseudo-CHL.

Given our case history and the literature on the subject, caution should be exercised in clinical practice when performing stapes surgery. Although the possibility of good audiological results after stapes surgery exists, the risk of partial or poor success must be considered, as only the component of HL related to stapedial plate stiffness can be resolved. 

4 out of 5 patients in our sample also have another factor against the indication for stapes surgery: the empty space around the cochlea can grow and connect with the cochlear lumen. This feature indicates communication between the CSF and perilymph. This is a very important aspect as this communication could lead to intraoperative gusher at the time of stapedotomy. This is a serious complication resulting in loss of perilymphatic fluid and CSF, which in some cases may lead to anacusis or significant HL [27]. 

Ultimately, we cannot be certain that cavitary lesions are able to induce pseudo-CHL, but we have collected some evidence to support this thesis. The most significant is related to the partial results obtained by stapes surgery: the incidence of ABG persistence in patients with pericochlear cavities is higher than in patients with noncavitary otosclerosis. In fact, stapes surgery is unable to suppress the third-window effect, this lasts into the postoperative phase. It is known that the third-window effect can induce vestibular disorders, but we recorded no vestibular symptoms in our sample. This can be an issue against our hypothesis. However, cavitating lesions involve only the anterior labyrinth, not vestibule and semicircular canals, which are deputed to balance.

In the future, it will be necessary to supplement the study by collecting new cases to verify the true impact of these cavities on the audiometric examination and surgical indications. Only a larger sample will allow the development of adequate statistical investigations that can guide physicians in defining surgical indications and audiological prognosis, and that can help patients set appropriate stapes surgical expectations.

## 5. Conclusions

Osteogenesis imperfecta and otosclerosis can cause retrofenestral spread of bony rarefaction; in rare cases, these conditions can lead to the formation of cavities around the cochlea in communication with the IAC. Sometimes the cavity communicates with the cochlear lumen. These patients develop pseudo-CHL, and stapes surgery should be carefully considered in view of the potential limited benefits and adverse effects.

## Figures and Tables

**Table 1:** Patients included in the study. PTA, Pure Tone Average (0.5–4 KHz); BC, Bone Conduction; OI, Osteogenesis Imperfecta; SSCD, Superior Semicircular Canal Dehiscence; MHL, Mixed Hearing Loss; CHL, Conductive Hearing Loss; SNHL, Sensorineural Hearing Loss; ABG, Air Bone Gap. (*) only the right ear data of this patient are reported as the left ear does not meet the study inclusion criteria.

Patient	Gender	Disease	Side	Communication Cavity-Cochlea	First Diagnosis					Progression					
					Age	Type of HL	PTA	BC PTA	Acoustic Reflex	Years after First Diagnosis	Type of HL	PTA	BC Threshold	Stape Surgery	Stapes Surgery— Outcome
**#1**	F	OI	R	Not present	20	MHL	55 dB	30 dB	Absent	27	SNHL	90 dB	85 dB	Performed	Improving
			L	Not present		MHL	50 dB	30 dB	Absent	27	SNHL	Anacusis	/	Performed	Persistent ABG
**#2**	M	OI	R	Present	23	MHL	65 dB	45 dB	Absent	8	MHL	90 dB	50 dB	Not Performed	/
			L	Present		MHL	70 dB	45 dB	Absent	8	MHL	70 dB	50 dB	Not Performed	/
**#3**	F	Otosclerosis	R	Present	40	CHL	30 dB	15 dB	Absent	18	MHL	60 dB	35 dB	Not Performed	/
		Otosclerosis -SSCD	L	Present		CHL	25 dB	15 dB	Absent	18	MHL	35 dB	20 dB	Not Performed	/
**#4**	M	Otosclerosis	R	Present	30	SNHL	50 dB	50 dB	Absent	12	SNHL	Anacusis	/	Not Performed	/
			L	Present		SNHL	60 dB	60 dB	Absent	12	SNHL	Anacusis	/	Not Performed	/
**#5**	F	Otosclerosis	R *	Present	23	CHL	30 dB	10 dB	Absent	5	MHL	75 dB	55 dB	Not Performed	/

## Data Availability

The data presented in this study are available on request from the corresponding author. The data are not publicly available because they are sensitive health data.
